# A higher TyG index level is more likely to have enhanced incidence of T2DM and HTN comorbidity in elderly Chinese people: a prospective observational study from the reaction study

**DOI:** 10.1186/s13098-024-01258-3

**Published:** 2024-01-30

**Authors:** Wanlu Su, Jie Wang, Kang Chen, Wenhua Yan, Zhengnan Gao, Xulei Tang, Qin Wan, Zuojie Luo, Guang Ning, Yiming Mu

**Affiliations:** 1 Department of Endocrinology, The First Clinical Medical Center of Chinese People’s Liberation Army General Hospital, No. 28 Fuxing Road, Beijing, 100853 China; 2 School of Medicine, Nankai University, No. 94 Weijin Road, Tianjin, 300071 China; 3 Department of Endocrinology, Affiliated Beijing Chaoyang Hospital of Capital Medical University, No. 8 Workers Stadium South Road, Chaoyang District, Beijing, 100853 China; 4 Department of Endocrinology, Dalian Municipal Central Hospital, No. 826 Southwest Shahekou District Road, Dalian, 116033 China; 5 Department of Endocrinology, The First Hospital of Lanzhou University, Lanzhou, Gansu China; 6 Department of Endocrinology, Center Hospital of Dalian, Dalian, Liaoning China; 7 Department of Endocrinology, Affiliated Hospital of Luzhou Medical College, No. 25 Taiping Road, Luzhou, 646000 China; 8 Department of Endocrinology, The First Affiliated Hospital of Guangxi Medical University, Nanning, Guangxi China; 9 State Key Laboratory of Medical Genomics, Department of Endocrinology, Shanghai National Research Center for Endocrine and Metabolic Disease, Shanghai Institute for Endocrine and Metabolic Disease, Ruijin Hospital, Shanghai Jiaotong University School of Medicine, Shanghai, China

**Keywords:** TyG index, T2DM, HTN, Elderly people

## Abstract

**Background:**

Triglyceride glucose index (TyG index) was related with both type 2 diabetes (T2DM) and hypertension (HTN). Prospective studies linking the TyG index to the incidence of T2DM and HTN comorbidity remain unclear. This study aimed to to explore the longitudinal association between TyG and new-onset T2DM with HTN.

**Methods:**

4,434 subjects (1249 males and 3185 females) without initial T2DM and HTN were followed up for 7 years. This study was conducted from November 2011 to August 2018 in the Gucheng, Laoshan and Jinding communities of Beijing. The incidence of T2DM with HTN during the 7-year follow-up was identified as the endpoint. The TyG index was divided into four quartiles: the < 25% level, the 25–50% level, the 50–75% level and the ≥ 75% level. The relationships between the TyG index and T2DM with HTN were evaluated by Cox proportional hazards regression models.

**Results:**

During 7 years, the augmented trend of T2DM with HTN was observed in the participants. After adjusting for all confounding factors, compared with those in the lowest quartile of TyG index, the population in the highest quartile of TyG index had a higher risk of T2DM with HTN (hazard ratio (HR), 2.878; 95% confidence intervals (95% CI), 1.230–6.731, P = 0.015), however, the association remained significant only in the female population (HR 2.753, 95% CI, 1.061–7.139, p = 0.037). The TyG had superior predictive ability of increased risk of T2DM with HTN for the populations of older age (≥ 65 years) (HR 2.694, 95% CI 1.212–5.989, p = 0.015), higher eGFR (≥ 90 mL/(min·1.73 m^2^)) (HR 2.603, 95% CI 1.164–5.818, p = 0.020) or obesity (BMI ≥ 28 kg/m^2^) (HR 2.547, 95% CI 1.001–6.478, p = 0.020).

**Conclusion:**

A population with a higher TyG index level was more likely to have an enhanced incidence of T2DM and HTN comorbidity. TyG index could have the significance of clinical in early protection against T2DM with HTN.

## Introduction

Diabetes mellitus (DM) has emerged as a major part of public health concern worldwide can be attributed to its high morbidity, related mortality and the rate of disability [1]. The number of diabetic patients in China currently ranks first in the world according to the diabetes atlas of the International Diabetes Federation [2]. DM is a well-known risk factor for any form of cardiovascular disease (CVD) [3] and the risk for CVD of subjects with type 2 diabetes (T2DM) is 2 ~ fourfold higher than those without [4]. Hypertension (HTN) is prevailing in the complications of diabetes [5] and constitutes a critical challenge to the health burden as it elevates the prevalence of CVD, and this is even further aggravated if patients with concomitant T2DM [6]. These studies have indicated that the coexistence of DM and HTN intensifies the progression of CVD.

The presence of dyslipidemia and glucose abnormality are related to increased risk for both DM and HTN [7,8]. It has since long been established that dyslipidemia which characterized by low levels of high-density lipoprotein cholesterol (HDL-C), and high levels of total cholesterol (TC), low-density lipoprotein cholesterol (LDL-C), the overproduction of triglycerides (TG), is the cornerstone of arteriolosclerosis and is able to increase prevalence of CVD in high-risk subjects such as those with HTN or DM [9–11]. It is well-known that statin medicines mediate the biggest benefit with CVD risk decrease, particularly among subjects with T2DM [12–14]. The findings suggest that glucose abnormality per se has an influence on HTN risk [15]. In addition, not only can the aggressive glycemic control lower blood pressure (BP) of ambulatory, but also these medications can be able to postpone the onset and to decrease the mortality of CVD that has important significance in improvement of certain clinical endpoints [16,17]. Similarly, it is reported that the insulin secretion abilities of patients with diabetes ameliorated owing to the appropriate treatment for high levels of fasting plasma glucose (FBG) and TG [8].

The reasonable pathophysiological mechanisms linking HTN, hyperglycemia, dyslipidemia and T2DM are regarded as insulin resistance (IR) and increased oxidative stress and visceral fat accumulation as well as elevated vascular inflammation [18–20]. Resent studies have shown that IR is regarded as a crucial mediator of HTN and CVD, and found that IR could be considered as the available risk identifier tool for the prevalence of DM and HTN [21]. In the researches, the glucose clamp technique is employed as the gold standard to assess IR and the insulin resistance (HOMA-IR) is used as the most commonly strategy to measure IR [22]. Nevertheless, in large epidemiological studies, all these techniques may have limitations owing to the prohibitive expense and low feasibility [23]. Thus, we need screening tools that are widely practical and affordable to indicate T2DM with HTN for the general population.

Some studies have suggested triglyceride glucose (TyG) index, the product of FBG and TG, as an ideal surrogate tool for IR [24]. The TyG index has shown higher specificity, sensitivity, time-efficient and cost-effective for identification of IR compared to other assessment indexes such as the hyper-insulinemic euglycemic clamp and HOMA-IR [24,25]. In addition, the TyG index has been implemented by clinicians of different countries to determine IR of diabetes and non-diabetes populations [26,27]. Moreover, this substitutional index has been validated as a reliable method to estimate CVD events and severity of coronary artery calcification [28]. Also, of importance was the TyG index which showed significant associations with coronary artery disease and HTN [29,30].

However, data are limited on the ability of the TyG index to predict HTN incidence with T2DM. The TyG index should be validated in the Chinese population. To address knowledge gap, the present study conducted a Chinese population-based prospective observational study of subjects without T2DM and HTN at baseline to explore whether the TyG index has the predictable capacity for a high risk of incident T2DM and HTN in adults in China.

## Methods

### Study subjects

This prospective observational study was conducted within the framework of a multicenter longitudinal REACTION study (Risk Evaluation of cAncers in Chinese diabetic Individuals), details of the baseline results and methods of the study have been described in a previous study [31]. The baseline study was conducted during November 2011 to August 2012 in Gucheng, Laoshan and Jinding communities of Beijing, and the subjects are 3-year intervals follow-up. Signed informed consents were obtained from all subjects before data collection. 193,14 individuals aged 40 years and over were registered by a cluster sampling method and underwent the baseline (first phase) examination. Because 12,219 subjects were excluded due to the following situations: previous history of diabetes and hypertension at baseline (n = 4484); subjects with T2DM at baseline [FBG ≥ 7.0 mmol/L, or 2 h post-load blood glucose (PBG) ≥ 11.1 mmol/L] (n = 1762); subjects with hypertension at baseline [systolic blood pressure (SBP) ≥ 140 mmHg or diastolic blood pressure (DBP) ≥ 90 mmHg] (n = 1877); using lipid-lowering drugs or with hyperlipidemia at baseline (n = 1,982); using antidiabetic-medication therapy at baseline (n = 925); using antihypertensive-medication therapy at baseline (n = 1022); missed data and/or included outliers (n = 167). Finally, a total of 7095 subjects were invited to undergo the examination of follow-up, and after excluding 346 subjects who had died during follow-up, 2315 subjects who didn’t attend any follow-up examination and 312 subjects who had used lipid-lowering drugs within the 7 years. We had 4122 subjects (1193 males and 2929 females) eligible for the present study (response rate 58.10%) and they were successfully enrolled in September 2018 to re-investigate the related information.

The protocol of this study was approved by the Committee on Human Research at Rui-Jin Hospital affiliated with the School of Medicine, Shanghai Jiao Tong University. This study was carried out by the Helsinki Declaration.

### Clinical data and biochemical indicators

Participants were required to have blood samples taken, take a 100 g steamed-bread meal test or 75 g oral glucose tolerance test (OGTT), undergo anthropometric measurements, and complete a standard questionnaire. The trained nurses collected the data from a standard questionnaire including lifestyle, family history, medical histories, sociodemographic characteristics and current medication use. The same trained nurses measured the waist circumference (WC), body weight, height, and before the measurements, the subjects were asked to take in light clothing without shoes. WC was surveyed at the horizontal level of the ligature’s midpoint between the inferior margin of the twelfth rib and the anterior superior spine. Body mass index (BMI) was calculated as weight divided by height squared (kg/m^2^). BP was measured three times consecutively at 1-min intervals after at least 5 min of rest in a sitting position, and the average of the three values of SBP and DBP was recorded. All participants were told to fast for at least 12 h before the blood samples were collected. Subjects with or without a history of diabetes took standard meals containing 100 g carbohydrates or a standard 75 g glucose solution. Sera were aliquoted into 0.5-mL Eppendorf tubes within 2 h after blood collection and shipped in dry ice at – 80 ° C to the central laboratory located at the Shanghai Institute of Endocrine and Metabolic Diseases. FBG, PBG, TG, LDL-C, HDL-C, TC, gamma-glutamyl transferase (GGT), aspartate aminotransferase (AST), alanine aminotransferase (ALT) and serum creatinine (Scr) were measured using standard enzymatic automated methods on an autoanalyzer (c16000 system, ARCHITECT ci16200 analyzer; Abbott Laboratories, Lake Bluff, IL) in the central laboratory. Haemoglobin A1c (HbA1c) was determined with a high-performance liquid chromatography method (HPLC, Variant II and D-10 Systems; Bio-Rad, Hercules, CA). The estimated glomerular filtration rate (eGFR) was calculated using the modified MDRD equation for the Chinese population: eGFR = 186 × (serum creatinine × 0.011) ^−1.154^ × (age)^−0.203^ × (0.742 if female) × 1.233 [32].

Current smoking status were defined as whether subjects smoked at least 7 per week or 1 cigarette per day regularly during the past half year, as well as current drinking intake were defined as whether subjects consumed alcohol once per week regularly during the past half year. Determination of CVD events were according to the self-report of participants, including history of stroke, myocardial infarction (MI) or coronary heart disease (CHD).

### Definition of variables

HTN was diagnosed as any self-reported history of HTN and/or regularly taking antihypertensive drugs, or SBP ≥ 140 mmHg, or DBP ≥ 90 mmHg. T2DM was diagnosed as any self-reported history of T2DM, and/or regular oral hypoglycemic agents or insulin use, or FBG ≥ 7.0 mmol/L, or PBG ≥ 11.1 mmol/L according to the WHO guidelines [33]. Data collections at follow-up were the same as for the baseline examination. TyG index was calculated as Ln [FBG (mg/dl) * TG (mg/dl)/^2^] [24]. And it was divided into four groups (quartile 1 to quartile 4): quartile 1(Q1), the < 25% group (For all subjects, For men, For women); quartile 2(Q2), the 25–49% group (For all subjects, For men, For women); quartile 3(Q3), the 50–74% group (For all subjects, For men, For women); quartile 4(Q4), the 75–100% group (For all subjects, For men, For women).

### Statistical analysis

SPSS version 24.0 (IBM, Chicago, IL, USA) was used to perform all statistical analyses. A two-sided P value of less than 0.05 was set as the significance level. Data were presented as means ± standard deviations (SD) for continuous variables with normal distribution and medians (Inter-Quartile Range, IQR), or n (%) for continuous variables without normal distribution, or categorical variables. The variance homogeneity test was analyzed by Brown Forsythe test. The significant differences in categorical and continuous variables, respectively, were compared using the chi-square test and the Mann–Whitney U test. Cox proportional hazard regression models were built to identify the risk of T2DM with HTN for four baseline TyG index groups. Non-adjusted model, controls for nothing, while adjusted I was adjusted for gender and age. Model II, further controlling for smoking habits, drinking habits; history of CVDs; age; gender; ALT; AST; heart rate; SBP; DBP; BMI; eGFR and HbA1c. The associations between the TyG index and T2DM with HTN were conducted in subgroups of age (< 55, 55–64, and ≥ 65 years), eGFR (< 60, 60–90, and ≥ 90 mL/min/1.73 m^2^) and BMI (< 18.5 kg/m^2^; 18.5–24 kg/m^2^; 24–28 kg/m^2^, BMI of ≥ 28 kg/m^2^). The present study explored the relationships between TyG index and the stratified variables among participants with enhanced risk of T2DM with HTN. The possible interactions of TyG index and stratified variables were explored after adjustment for sex, history of CVDs; SBP; DBP; ALT; AST; heart rate; HbA1c; smoking habits, drinking habits and age or BMI or eGFR. P < 0.05 (two-taile) was regarded as statistically significant.

## Results

### Basic characteristics of T2DM and HTN

The present longitudinal study was conducted for 7 years to explore the prophetic ability of the TyG index for the prevalence of T2DM with HTN. The basic clinical and demographic characteristics of the participants were shown in Table 1. This current research finally enrolled 4122 subjects, and T2DM with HTN developed in 67 subjects (31 men and 36 women). The categorical variables were compared using the chi-square test (sex, smoking status, drinking intake and CVD events), and the continuous variables were compared using the Mann–Whitney U test (TyG, age, BMI, SBP, DBP, heart rate, FBG, PBG, HbA1c%, ALT, AST, GGT, TG, TC, LDL-C, HDL-C and eGFR). Notably, comparing with participants with T2DM and HTN, the TyG level of non-T2DM with HTN subjects were significantly lower, as well as younger, had lower SBP and DBP, lower level of FBG and PBG, a less favorable metabolic profile (TG, TC, LDL-C, GGT, AST, ALT, HbA1c), and a lower frequency of CVD. In addition, the levels of heart rate, HDL-C and eGFR, frequent drinkers and smokers all tended to increase in the non-T2DM with HTN group (P < 0.001).

**Table 1:** Characteristics of study population by T2DM with HTN

	No T2DM with HTN	T2DM with HTN	P value
N	4055	67	<
TyG(2011)	7.70(7.16–8.03)	8.83(8.37–9.36)	< 0.001
Age,years	54.00(49.00–58.00)	55.50(50–61.25)	< 0.001
BMI,kg/m^2^	24.61(22.62–26.78)	25.29(23.87–27.65)	< 0.001
SBP,mmHg	121.93(114.33–129.33)	128.48(118.17–134.83)	< 0.001
DBP,mmHg	72.83(66.67–78.39)	73.67(69.25–79.67)	< 0.001
Heart rate	73.00(67.00–80.00)	71.00(65.00–76.50)	< 0.001
FBG,mmol/L	5.34(4.97–5.60)	5.67(5.26–6.36)	< 0.001
PBG,mmol/L	6.49(5.52–7.49)	8.45(6.54–9.37)	< 0.001
HbA1c,%	5.70(5.50–6.00)	5.90(5.60–6.20)	< 0.001
ALT,U/L	15.98(13.00–22.20)	18.40(15.00–23.15)	< 0.001
AST,U/L	19.13(16.50–22.30)	18.50(16.65–21.45)	< 0.001
GGT,U/L	18.32(13.60–25.30)	19.70(15.25–29.05)	< 0.001
TG,mmol/L	1.21(0.84–1.63)	1.39(0.94–2.27)	< 0.001
TC,mmol/L	4.87(4.50–5.35)	5.13(4.55–5.75)	< 0.001
LDL-C,mmol/L	3.19(2.66–3.68)	3.08(2.60–3.63)	< 0.001
HDL-C,mmol/L	1.43(1.23–1.71)	1.27(1.07–1.46)	< 0.001
eGFR,mL/(min·1.73 m^2^)	123.87(110.33–137.12)	119.85(107.16–135.84)	< 0.001
Sex, (%)			
Male	1162(28.66%)	31(46.27%)	
Female	2893(71.34%)	36(53.73%)	< 0.001
Current smoker, no. (%)	578(14.25%)	8(11.94%)	< 0.001
Past smoker, no. (%)	226(5.57%)	1(1.49%)	< 0.001
Current alcohol drinker, no. (%)	1189(29.32%)	16(23.89%)	< 0.001
Past alcohol drinker, no. (%)	879(21.68%)	14(20.90%)	< 0.001
CVDs, (%)			< 0.001
Yes	136(3.35%)	3(4.48%)	
No	3919(96.65%)	64(95.52%)	< 0.001

Data were mean ± SD or median (IQR) for skewed variables or numbers (proportions) for categorical variables

HTN: hypertension; T2DM: type 2 diabetes. BMI: body mass index; SBP: systolic blood pressure; DBP: diastolic blood pressure; heart rate; FBG: fasting plasma glucose; PBG: 2 h post-load blood glucose; HbA1c: glycosylated hemoglobin; ALT: alanine transferase; AST: aspartate transferase; GGT: gamma-glutamyl transferase; TG: triglyceride; TC: high cholesterol; LDL-C: low-density lipoprotein cholesterol; HDL-C: high-density lipoprotein cholesterol; eGFR: lower estimated glomerular filtration rate; CVD: cardiovascular disease

*Significant data (*P* < 0.05)

### Association between the TyG index and incident T2DM with HTN

To evaluate separately the ability of the TyG index and its components to predict the incident T2DM with HTN, we constructed multiple logistic regression models. Table 2 showed that the risk of T2DM with HTN according to the baseline TyG index in all subjects were significant only in the fourth quintiles of the TyG index after further adjustments (HR 2.821, 95% CI 1.210–6.897, p = 0.015). As shown in Table 3 which subjects separated into men and women, we found that the fourth quartile of the TyG index (in men: HR 2.126, 95% CI 1.033–4.419, p = 0.032; in women: HR 2.259, 95%CI 1.181–4.904, p = 0.006) was associated with the incident T2DM with HTN in the unadjusted model. However, after further adjustment, the association between the fourth quartile TyG index and T2DM with HTN was only significant in women (HR 2.736, 95% CI 1.098–7.235, p = 0.036).

**Table 2:** Risk of T2DM with HTN according to baseline TyG in unadjusted and adjusted models in all subjects

Variable	Non-adjusted	Adjusted model I	Adjusted model II
	HR(95%CI) P-value	HR(95%CI) P-value	HR(95%CI)P-value
TyG in transform Q4			
Q1	1	1	1
Q2	2.102(0.978–4.702) 0.061	1.987(0.879–4.364) 0.081	1.788(0.753–4.120) 0.169
Q3	1.279(0.498–3.112) 0.509	1.266(0.434–3.112) 0.585	0.718(0.377–1.521) 0.414
Q4	3.120(1.864–7.247) 0.002*	3.112(1.439–6.371) 0.003*	2.821(1.210–6.897) 0.015*

Non-adjusted: Adjusted for no confounding factors;

adjusted model I: Adjusted for age and gender;

adjusted model II: age; gender; diabetic family history; history of CVDs; SBP; DBP; BMI; ALT; AST; heart rate; eGFR; HbA1c; smoking habits, drinking habits

*Significant data (*P* < 0.05)

**Table 3:** Risk of T2DM with HTN according to baseline TyG in unadjusted and adjusted models in all subjects by gender

Variable	Non-adjusted	Adjusted model I	Adjusted model II
	HR(95%CI) P-value	HR(95%CI) P-value	HR(95%CI)P-value
TyG in transform Q4			
Men (n = 1193)			
Q1	1	1	1
Q2	2.776(0.724–10.023) 0.108	2.651(0.793–10.129) 0.121	2.229(0.524–8.592) 0.229
Q3	1.182(0.398–3.209) 0.867	1.142(0.617–3.276) 0.790	0.873(0.221–2.467) 0.918
Q4	2.126(1.033–4.419) 0.032*	2.208(1.004–4.367) 0.025*	1.544(0.608–3.354) 0.202
Women (n = 2929)			
Q1	1	1	1
Q2	1.711(0.710–4.524) 0.219	1.689(0.988–4.234) 0.383	1.594(0.245–5.987) 0.843
Q3	0.658(0.912–2.001) 0.543	0.732(0.210–1.369) 0.439	0.830(0.126–1.890) 0.315
Q4	2.259(1.181–4.904) 0.006*	2.568(1.426–4.376) 0.011*	2.736(1.098–7.235) 0.036*

Non-adjusted: Adjusted for no confounding factors;

adjusted model I: Adjusted for age;

adjusted model II: age; diabetic family history; history of CVDs; SBP; DBP; BMI; ALT; AST; heart rate; eGFR; HbA1c; smoking habits, drinking habits

*Significant data (*P* < 0.05)

### Association between the TyG index and incident T2DM with HTN in all subjects with LDL-C < 2.6 mmol/L or HDL-C ≥ 1.0 mmol/L

All subjects were categorized into two groups, HDL-C ≥ 1.0 mmol/L and LDL-C < 2.6 mmol/L, according to the Chinese guideline for the management of dyslipidemia (revised in 2016) [34]. As shown in Table 4, the higher level of TyG index (the fourth quartile) were obvious significantly associated with the incident T2DM with HTN though HDL-C or LDL-C was well controlled (well-controlled level of HDL-C: HR 2.809, 95% CI 1.354–6.111, p = 0.021. well-controlled level of LDL-C: HR 2.907, 95% CI 1.223–6.309, p = 0.019).

**Table 4:** Risk of T2DM with HTN according to baseline TyG in unadjusted and adjusted models in all subjects with LCL-C < 2.6 mmol/L or HDL-C > 1.0 mmol/L

Variable	Non-adjusted	adjusted model I	adjusted model II
	HR(95%CI) P-value	HR(95%CI) P-value	HR(95%CI)P-value
All subjects			
**HDL-C > 1.0 mmol/L**			
TyG in transform Q4			
Q1	1	1	1
Q2	2.193(0.894–4.379) 0.075	1.762(0.801–4.241) 0.078	2.223(0.894–5.009) 0.069
Q3	1.562(0.590–3.223) 0.736	1.344(0.520–2.872) 0.676	1.290(0.369–2.901) 0.729
Q4	3.751(1.442–6.374) 0.003*	2.371(1.845–6.326) 0.005*	2.809(1.354–6.111) 0.021*
**LDL-C < 2.6 mmol/L**			
TyG in transform Q4			
Q1	1	1	1
Q2	2.223(0.893–4.221) 0.059	2.053(0.953–4.469) 0.085	2.129(0.879–4.156) 0.069
Q3	1.529(0.346–3.469) 0.307	1.512(0.580–3.273) 0.542	1.109(0.389–2.815) 0.815
Q4	3.414(1.582–7.733) 0.001*	3.818(1.538–7.029) 0.002*	2.907(1.223–6.309) 0.019*

Non-adjusted: Adjusted for no confounding factors;

adjusted model I: Adjusted for age and gender;

adjusted model II: Adjusted for diabetic family history; history of CVDs; SBP; DBP; BMI; ALT; AST; heart rate; eGFR; HbA1c; smoking habits, drinking habits

*Significant data (*P* < 0.05)

### Association between the TyG index and incident T2DM with HTN for stratified subgroups of age, BMI and eGFR

As shown in Table 5, to further validate the associations of the different subgroups between the TyG index and incident T2DM with HTN, the stratified analyses were performed after further adjusting with sex, history of CVDs; SBP; DBP; ALT; AST; heart rate; HbA1c; smoking habits, drinking habits and age or BMI or eGFR. Our study found that compared with those with lower TyG index levels, participants with higher TyG index levels (the Q4) were more significantly associated with T2DM with HTN in the older age, higher level of BMI and both eGFR subgroups. It’s worthy that the associations were more markedly significant in the participants of the higher TyG index (the Q4) with the oldest age (≥ 65 years) (HR 2.539, 95% CI 1.227–5.863, p = 0.017), higher eGFR (≥ 90 mL/(min·1.73 m^2^)) (HR 2.876, 95% CI 1.603–5.886, p = 0.020) or obesity (BMI ≥ 28 kg/m^2^) (HR 2.767, 95% CI 1.023–6.778, p = 0.023). Interactions between the TyG index and stratified variables were not found.

**Table 5:** Risk of T2DM with HTN according to baseline TyG in unadjusted and adjusted models for different levels of age, BMI, eGFR

Variable	TyG Q1	TyG Q2		TyG Q3		TyG Q4		P for interaction
	Reference	HR(95%CI)	*P* value	HR(95%CI)	*P* value	HR(95%CI)	*P* value	
Age, year ^**a**^								0.528
< 55	1	N		N		N		
55–64	1	N		N		N		
≥ 65	1	2.109(0.788–4.624)	0.151	1.279(0.412–2.910)	0.677	2.539(1.227–5.863)	0.017*	
eGFR, ml/min per 1.73m^2 d^								0.907
eGFR ≥ 90	1	2.040(0.698–4.700)	0.089	1.231(0.902–2.468)	0.932	2.876(1.603–5.886)	0.020*	
60 ≤ eGFR < 90	1	1.490(0.160–3.724)	0.191	1.476(0.229–2.473)	0.259	1.490(0.162–1.882)	0.819	
eGFR < 60	1	1.443(0.769–1.994)	0.732	1.102(0.367–1.909)	0.324	1.909(0.947–3.314)	0.635	
BMI, kg/m^2 e^								0.812
BMI < 18.5 (n = 1117)	1	N		N		N		
18.5 ≤ BMI < 24 (n = 18097)	1	0.919(0.828–1.234)	0.527	1.321(0.365–1.098)	0.321	1.102(0.954–1.276)	0.057	
24 ≤ BMI < 28 (n = 15669)	1	0.533(0.468–2.179)	0.790	0.499(0.281–1.436)	0.129	1.763(0.528–3.110)	0.455	
BMI ≥ 28 (n = 5791)	1	0.523(0.119–2.176)	0.431	0.153(0.008–2.332)	0.448	2.767(1.023–6.778)	0.023*	

*Significant data (*P* < 0.05)

a Age subgroup: adjusted for sex, diabetic family history; history of CVDs; SBP; DBP; BMI; ALT; AST; heart rate; eGFR; HbA1c; smoking habits, drinking habits

b eGFR subgroup: adjusted for age, sex, diabetic family history; history of CVDs; SBP; DBP; BMI; ALT; AST; heart rate; HbA1c; smoking habits, drinking habits

c BMI subgroup: adjusted for age, sex, diabetic family history; history of CVDs; SBP; DBP; eGFR; ALT; AST; heart rate; HbA1c; smoking habits, drinking habits

## Discussion

For all we know, our present study is the first prospective study to explore the associations between the TyG index and T2DM with HTN in a Chinese general population. After the adjustment of potential compounders, the present study showed that the highest TyG index level (Q4) was associated with a higher incidence of T2DM and HTN comorbidity during a follow-up of 7 years, and it indicated that the TyG index was an independent predictor of new-onset T2DM with HTN, especially, it was more significant in females after adjusting the confounding factors. In the further stratified analyses, we found that subjects who were old (≥ 65), obesity (BMI ≥ 28 kg/m^2^) or those with normal eGFR (≥ 90 mL/ (min·1.73 m^2^) with higher TyG index level were more likely to have increased risk of T2DM with HTN than those with lower TyG index level. Therefore, we could pay more attention to the TyG index which is vital for the early detection and prevention of new-onset T2DM with HTN of the specific population in clinical practice, and people with higher TyG index levels would be aware of the increased risk, to change lifestyle at early stages.

Recently, the TyG index has been of enhanced interest. A study demonstrated that compared with the homeostatic model assessment and the hyper-insulinemic euglycemic clamp, the TyG index has high specificity and sensitivity for the diagnosis of insulin resistance. [35] Our previous study reported that the TyG index was associated with HTN in a general population based on a cross-sectional design [36]. Previous studies have concluded that elevated TyG index indicated several CVD and vascular damage [37,38]. This index have a large possibility to easily apply for the early detection of IR, arterial stiffness which is a severe adverse event in the HTN subjects and other diseases in clinical practice [39]. All the above studies demonstrated that the TyG index is an effective biomarker for identifying metabolic diseases in the future. We speculate the potential point of mechanisms about the effect of the TyG index to indicate the risk of T2DM with HTN may be IR. Generally, for metabolic disease, IR plays an important role and is a crucial element of increased risk of HTN [40]. IR could lead to various levels of oxidative responses and the impair function of endothelial [41], resulting in a low-level state of inflammation [42]. Additionally, one crucial factor of progressing to arterial stiffness was inflammation, and became a key role involved in the pathogenesis of HTN development [43]. Meanwhile, the fact that the association between the TyG index and IR was significant has been universally recognized [44].

Moreover, the finding about a sex difference of the present study implied that the association between the TyG index and T2DM with HTN was more significant in female compared with male, which means that sex-specific hormones of elderly people could partly lead to T2DM with HTN progression. Although poorly clarified, there are possible crucial pathways that may link the TyG index and T2DM with HTN. Since hormonal alterations could influence fat redistribution in perimenopausal or postmenopausal females, the enhanced accumulation of visceral fat and adiposity owing to decreased oestrogen levels could lead an increased risk of inflammation progression [45,46], and inflammation likely increases the risk of metabolic abnormalities such as T2DM or HTN. Interestingly, the present study showed that the association was more remarkable in older subjects rather than younger subjects. In a previous study, increased age was reported to accompany reduced body height/weight, increased fat mass and redistributed visceral adipose [47]. Furthermore, longer exposure to the environment might account for the discrepancies between age levels, and elderly females were observed to have higher BP than males [48].

One of the strengths of this study was that it was a prospective cohort study to confirm the relationship of the TyG index and T2D with HTN in the general China population after the adjustment of confounding factors. Besides, the present study used the 2-h OGTT to diagnose diabetes cases, so the reliability of the findings is enough. However, there also were some limitations: (1) the present study was only based on middle-aged and elderly people in China and the findings couldn’t be generalized to other populations. (2) the unavoidable bias of the long-term cohort study which was caused by the loss of follow-up existed. (3) although we considered the use of antihypertensive, lipid-lowering and anti-hypoglycaemic drugs, there was still a possibility that other medications may influence the association between the TyG index and T2DM with HTN. Herein, in routine clinical practice, the fact should be emphasized that the TyG index was a more convenient, efficient tool to identify the potential population at high risk of T2DM with HTN.

## Conclusion

In conclusion, this study confirms that the TyG index is closely related to the occurrence of T2DM combined with HTN in obese and older (≥ 65) Chinese individuals with normal eGFR value, and the TyG index as a novel and simple and practical biological indicator have clinical significance in the new-onset identification of population with increased risk of T2DM with HTN. Based on the TyG index, a strategy can be developed to actively intervene to prevent the occurrence of T2DM combined with HTN.

## Data Availability

The datasets used to support this study are not freely available to protect the privacy of participants.

## Abbreviations

AST: Aspartate transaminase
ALT: Alanine transaminase
BMI: Body mass index
BP: Blood pressure
BG: Blood glucose
CI: Confidence interval
CKD: Chronic kidney disease
CVD: Cardiovascular disease
DBP: Diastolic blood pressure
eGFR: Estimated glomerular filtration rate
FBG: Fasting blood glucose
GGT: Gamma-glutamyl transferase
GH: Glomerular hyperfiltration
HbA1c: Glycosylated haemoglobin
HDL-C: High-density lipoprotein cholesterol
IQR: Interquartile range
IR: Insulin resistance
LDL-C: Low-density lipoprotein cholesterol
TyG: TRIGLYCERIDE glucose index
HR: Hazard ratio
PBG: 2 H post-load blood glucose
RHR: Resting heart rate
SBP: Systolic blood pressure
TC: Total cholesterol
TG: Triglycerides
T2DM: Type 2 diabetes mellitus
T2DM: Type 2 diabetes
HTN: Hypertension
WC: Waist circumference
